# Association of Caregiver Depression Risk With Patient Outcomes in Parkinson Disease

**DOI:** 10.1001/jamanetworkopen.2023.27485

**Published:** 2023-08-11

**Authors:** Rudmila Rashid, Whitley W. Aamodt, Sarah Horn, Nabila Dahodwala

**Affiliations:** 1 Department of Neurology, Perelman School of Medicine at the University of Pennsylvania, Philadelphia; 2 Department of Neurology, The University of Texas Health Science Center at San Antonio, San Antonio; 3 Leonard Davis Institute of Health Economics, University of Pennsylvania, Philadelphia, Pennsylvania

## Abstract

**Importance:**

Caregivers are integral to Parkinson disease (PD) care, but little information exists regarding how caregivers impact patient outcomes.

**Objective:**

To assess the association between caregivers reporting depression symptoms and patient quality of life (QOL), emergency department (ED) visits, and hospitalizations.

**Design, Setting, and Participants:**

This retrospective cohort study was conducted at 15 Parkinson’s Foundation Centers of Excellence within the US. The Parkinson’s Foundation Parkinson Outcomes Project registry was used to collect baseline data from January 1, 2016, to December 31, 2018, with subsequent annual study visits through July 31, 2020. Data were analyzed from August 5, 2020, to June 9, 2023. A convenience sample of 454 patients with PD and their caregivers was recruited during routine clinical visits with movement disorder specialists. Patients with a physician diagnosis of idiopathic PD who lived at home and had 1 or more follow-up study visits were included.

**Exposure:**

Caregiver depression symptoms using the Center for Epidemiologic Studies Depression Scale.

**Main Outcomes and Measures:**

Patient health-related QOL (measured by the 39-item Parkinson Disease Questionnaire), number of annual ED visits, and number of annual hospitalizations were measured. The independent association between caregivers reporting depression symptoms and patient outcomes was assessed using linear mixed-effects and Poisson regression models. The a priori hypotheses were that a greater number of depression symptoms reported via the Center for Epidemiologic Studies Depression Scale would be associated with worse patient QOL and a greater number of ED visits and hospitalizations.

**Results:**

Among 454 patient-caregiver dyads (patients: mean [SD] age, 67.3 [8.4] years; 320 men [70.5%]; caregivers: mean [SD] age, 65.9 [8.7] years; 326 women [71.8%]), the mean (SD) follow-up was 2.0 (1.4) study visits. Greater depression symptoms among caregivers were associated with worse patient QOL as measured by the Parkinson Disease Questionnaire (mean [SD] score, 33.78 [17.71], on a scale of 0-100, with higher scores indicating worse QOL, among patients with caregivers who had depression symptoms vs 24.50 [14.19] among patients with caregivers who did not have depression symptoms; β = 0.43; 95% CI, 0.28-0.58; *P* < .001) and more annual ED visits (β = 0.02; 95% CI, 0 to 0.04; *P* = .03) but not more hospitalizations (β = 0.02; 95% CI, −0.01 to 0.03; *P* = .10).

**Conclusions and Relevance:**

In this cohort study, patients with PD who had caregivers at higher risk of depression were more likely to have worse QOL and higher ED use than patients who had caregivers not at higher risk of depression. Additional caregiving resources and interventions to reduce caregiver depression symptoms could potentially improve patient outcomes.

## Key Points

**Question:**

What is the association between caregiver depression risk and patient outcomes in Parkinson disease?

**Findings:**

In this cohort study of 454 patient-caregiver dyads seen at Parkinson’s Foundation Centers of Excellence, patients whose caregivers reported greater depression symptoms were significantly more likely to have worse health-related quality of life and greater emergency department use but not hospitalizations.

**Meaning:**

These findings suggest that provision of additional resources and interventions to reduce caregiver depression symptoms is important to potentially improve outcomes among patients with Parkinson disease.

## Teaser

This cohort study assesses the association between caregiver depression symptoms and quality of life, emergency department visits, and hospitalizations among patients with Parkinson disease.

## Introduction

Caregivers are an invaluable resource who support the physical and emotional needs and provide day-to-day care for people with Parkinson disease (PD).^1,2^ Caregiver presence is associated with additional benefits, including more informative health care visits, delayed nursing home placement, and reduced health care costs.^3,4,5^

However, because of the physical, financial, and emotional investment caregiving requires, this role can have negative consequences for caregivers’ well-being.^6^ Research has revealed that caregiver burden is correlated with greater motor symptoms, cognitive impairment, disrupted sleep, and lack of reciprocity (also known as mutuality or the sharing of feelings and action in a relationship) among persons with PD.^7^ Despite these data, a substantial gap in knowledge exists regarding the eventual implications of caregiver burden for the prognosis of a patient with PD; this gap limits our understanding of potentially modifiable factors that could improve outcomes in PD. Therefore, this study aimed to examine the association of caregiver burden with longitudinal patient outcomes. We hypothesized that when caregivers reported greater depression symptoms, patients with PD would have worse health-related quality of life (QOL) and greater health care use.

## Methods

### Study Design and Sample

We conducted a retrospective cohort study using data from the Parkinson’s Foundation (PF) Parkinson Outcomes Project (PF-POP), a longitudinal cohort study of health outcomes among patients with PD. The PF-POP collects data annually from patients during their regularly scheduled clinical visits at 21 PF Centers of Excellence.^8^ Participants in the PF-POP were recruited as a convenience sample. Any patient who received PD care at a Center of Excellence participating in the PF-POP was offered the opportunity to enroll in the database, and no initial exclusions were made based on age, disease severity, or cognitive ability.^9^ The present study included patients and their caregivers enrolled in the PF-POP who completed a caregiver substudy between January 1, 2016, and December 31, 2018; this caregiver study involved a 1-time optional survey administered to caregivers at 15 PF sites located throughout the US. Patients and caregivers were recruited during routine clinical visits with movement disorder specialists. Data from up to 4 follow-up visits were used. To be included, data from the follow-up visits had to be collected through July 31, 2020. Data were analyzed from August 5, 2020, to June 9, 2023. This study was approved by the institutional review boards of each of the 15 sites. Written informed consent was obtained from both patients and caregivers; in cases in which a patient lacked the capacity to consent, written consent from a legal guardian was obtained along with patient assent. This study followed the Strengthening the Reporting of Observational Studies in Epidemiology (STROBE) reporting guideline for cohort studies.^10^

The term *caregiver* was defined using the International Organization for Standardization definition as any person providing care to a care recipient.^11^ Within our study, all caregivers included were nonprofessional (ie, not financially compensated) and either family members or friends of the patient with PD. This subset of caregivers is usually referred to as informal caregivers or family or friend caregivers; however, for brevity, *caregivers* was used in this article. All included patients were living at home and had a clinical diagnosis of idiopathic PD and at least 1 follow-up visit.

Overall, 663 patient-caregiver dyads participated in the PF-POP caregiver substudy. Of those, 209 patient-caregiver dyads were excluded because the patient was missing follow-up data (n = 183), the patient had a first follow-up study visit more than 2 years after the baseline visit (n = 23), or the caregiver was missing depression data (n = 3). The remaining 454 patient-caregiver dyads (68.5%) who had at least 1 follow-up visit within 2 years after the baseline visit and complete depression data were included.

### Measures

Data collected from patients included demographic characteristics, disease duration, comorbidities, medications, scores on delayed 5-word recall (range 0-5, with higher scores indicating better memory function) and verbal fluency (range 0 to no maximum, with higher scores indicating better verbal fluency) asessments, and scores on the 39-item Parkinson Disease Questionnaire (PDQ-39). The PDQ-39 is a self-report questionnaire measuring health-related QOL (range, 0-100, with higher scores indicating worse QOL).^12^ Clinical data for the patient also included Hoehn and Yahr stage, a disease stage rating scale that describes motor deficits in patients with PD (range, 1-5, with higher scores indicating more severe motor deficits).^13^ The scale focuses on unilateral vs bilateral disease and postural reflex impairment and can be used regardless of whether patients are receiving dopaminergic therapy.

Data collected from caregivers at the baseline visit only included demographic characteristics and scores on the Center for Epidemiologic Studies Depression Scale (CES-D), a self-report 4-factor, 20-item measure that aids in identifying individuals at risk for depression.^14^ Scores range from 0 to 60, and a score higher than 16 indicates a substantially higher risk of clinical depression; in this article, we referred to the subset of caregivers with CES-D scores higher than 16 as those at higher risk of depression or as those with depression symptoms. Caregiver demographic characteristics included race and ethnicity. Individuals classified themselves into racial and ethnic categories that were determined by the PF-POP (Asian, Black or African American, White, and multiple races for race and Latino vs non-Latino for ethnicity); race and ethnicity data were collected to characterize the caregiver sample more thoroughly. Race and ethnicity data were incomplete for the participants with PD.

Primary outcomes included each patient’s QOL (measured by overall PDQ-39 score), number of self-reported hospitalizations, and number of self-reported emergency department (ED) visits. These data were collected annually at baseline and all follow-up visits.

### Statistical Analysis

First, we compared both patient and caregiver characteristics between patients with a caregiver who had a CES-D score higher than 16 (and were thus at a higher risk of clinical depression) and patients with a caregiver who had a CES-D score less than or equal to 16 at baseline. In our analyses, we treated CES-D scores as both a categorical variable (score >16 or ≤16) and a continuous variable within the 2 groups. The 2 respective groups were compared at baseline using *t* tests for normally distributed continuous variables, Wilcoxon rank sum tests for skewed continuous variables, and χ^2^ tests for categorical variables. Specifically, when comparing caregiver demographic characteristics, we used a *t* test for means and SDs, a Wilcoxon rank sum test for medians and IQRs, and a χ^2^ test for categorical variables unless a value in a cell was less than 5, in which case we used a Fisher exact test.

Next, we used 3 mixed-effects linear and Poisson repeated-measures regression analyses via the maximum likelihood method to estimate the independent association between caregivers at higher risk of depression and (1) QOL (linear model), (2) hospitalizations (Poisson model), and (3) ED visits (Poisson model). Each outcome was the dependent variable for each model, which accounted for repeated observations within participants as random effects and controlled for patient age, sex, disease duration, Hoehn and Yahr stage, and cognitive measures as well as caregiver financial status and self-reported health. The statistical significance level was set at 2-tailed *P* = .05 Analyses were conducted using Stata software, version 16 (StataCorp LLC). A post hoc power analysis was conducted to test the detectable difference between 2 groups given the sample size with probability (power) of 0.80 and α = .05. This analysis revealed a detectable difference of 0.32 for mean ED visits and 0.29 for mean hospitalization rates.

## Results

Among 454 patient-caregiver dyads, the mean (SD) age of patients was 67.3 (8.4) years, while caregivers were slightly younger, with a mean (SD) age of 65.9 (8.7) years. Patients were more likely to be men (320 [70.5%] vs 134 women [29.5%]), while caregivers were more likely to be women (326 [71.8%] vs 128 men [28.2%]). Most caregivers were of White race (435 of 447 [97.3%]) and non-Latino ethnicity (384 of 393 [97.7%]). Patients had high educational levels, with 275 of 434 (63.4%) having a bachelor’s or graduate degree. Mean (SD) follow-up was 2.0 (1.4) annual visits.

Next, we compared the baseline characteristics of patients by whether they had caregivers with or without depression symptoms (Table 1). A total of 356 patients had caregivers without a higher risk of depression (CES-D score ≤16), and 98 had caregivers with a higher risk of depression (CES-D score >16). Among patients overall, the mean (SD) CES-D score was 9.8 (7.7), with scores of 6.6 (4.5) among the group with caregivers who did not screen positive for depression vs 21.6 (4.8) among the group with caregivers who screened positive (*P* < .001). There was a larger proportion of female patients in the group who had caregivers without a higher risk of depression (116 [ 32.6%]) compared with the group who had caregivers with a higher risk of depression (18 [18.4%]; *P* = .006). Patients who had caregivers without depression symptoms also had higher verbal fluency scores (mean [SD], 18.6 [6.7]) compared with patients who had caregivers with depression symptoms (mean [SD], 17.0 [6.2]; *P* = .03).

**Table 1.** Characteristics of Patients With Parkinson Disease and Caregivers by Caregiver Depression Symptoms

Characteristic	Participants, No./total No. (%)^a^		*P* value
	Caregiver without higher risk of depression (n = 356)^b^	Caregiver with higher risk of depression (n = 98)^c^	
**Patients**			
Age, mean (SD), y	66.9 (8.6)	68.5 (7.9)	.11
Sex			
Female	116/356 (32.6)	18/98 (18.4)	.006
Male	240/356 (67.4)	80/98 (81.6)	
Educational level			
Less than high school	2/343 (0.6)	0	.37
High school graduate	55/343 (16.0)	13/91 (14.3)	
Some college or associate’s degree	73/343 (21.3)	16/91 (17.6)	
Bachelor’s degree	99/343 (28.9)	36/91 (39.6)	
Graduate degree	114/343 (33.2)	26/91 (28.6)	
Disease duration, mean (SD), y	10.1 (5.5)	11.3 (5.2)	.06
Hoehn and Yahr stage, median (IQR)^d^	2 (2-3)	2 (2-3)	.07
Verbal fluency score, mean (SD)^e^	18.6 (6.7)	17.0 (6.2)	.03
Delayed 5-word recall score, median (IQR)^f^	4 (3-5)	4 (3-5)	.15
No. of comorbidities, median (IQR)	1 (0-2)	1 (0-2)	.92
No. of medications, median (IQR)	1 (0-2)	1 (0-2)	.30
**Caregivers**			
Age, mean (SD), y	65.6 (8.9)	67.1 (8.2)	.15
Sex			
Female	245/356 (68.8)	81/98 (82.7)	.007
Male	111/356 (31.2)	17/98 (17.3)	
Ethnicity			
Latino	2/83 (2.4)	7/310 (2.3)	.93
Non-Latino	81/83 (97.6)	303/310 (97.7)	
Race			
Asian	3/351 (0.9)	1/96 (1.0)	.88
Black or African American	5/351 (1.4)	1/96 (1.0)	
White	341/351 (97.2)	94/96 (97.9)	
Multiple races	2/351 (0.6)	0	
Financial status			
Some money left over	303/350 (86.6)	71/97 (73.2)	.005
Just enough to make ends meet	43/350 (12.3)	23/97 (23.7)	
Not enough to make ends meet	4/350 (1.1)	3/97 (3.1)	
Married or in a domestic partnership	347/356 (97.5)	95/98 (96.9)	.77
Self-reported health status			
Excellent	85/355 (23.9)	4/98 (4.1)	<.001
Very good	162/355 (45.6)	39/98 (39.8)	
Good	91/355 (25.6)	41/98 (41.8)	
Fair or poor	17/355 (4.8)	14/98 (14.3)	
Relationship to patient with PD			
Spouse or partner	333/355 (93.8)	94/97 (96.9)	.63
Child	13/355 (3.7)	2/97 (2.1)	
Other relative or friend	9/355 (2.5)	1/97 (1.0)	
Lives with patient with PD	331/354 (93.5)	93/97 (95.9)	.48
CES-D score, mean (SD)	6.6 (4.5)	21.6 (4.8)	<.001

Abbreviations: CES-D, Center for Epidemiological Studies Depression Scale; PD, Parkinson disease.

^a^ For comparisons of continuous variables, 2-sample *t* tests were used when means were reported, and Wilcoxon rank sum tests were used when medians were reported. For comparisons of categorical variables, χ^2^ and Fisher exact tests (for comparisons with values <5) were used. Smaller denominators reflect missing data.

^b^ CES-D score of 16 or lower on a scale of 0 to 60.

^c^ CES-D score of higher than 16 on a scale of 0 to 60.

^d^ Scored from 1 to 5, with higher scores indicating more severe motor deficits.

^e^ Scored from 0 to no maximum, with higher scores indicated better verbal fluency.

^f^ Scored from 0 to 5, with higher scores indicated better memory function.

Among caregivers, the group with depression symptoms had a higher proportion of women (81 [82.7%]) than the group without depression symptoms (245 [68.8%]; *P* = .007). The group with vs without depression symptoms also had a higher proportion of those who reported their financial status as not having enough to make ends meet (3 of 97 [3.1%] vs 4 of 350 [1.1%]; *P* = .005) and those who self-reported their health status as fair or poor (14 of 98 [14.3%] vs 17 of 355 [4.8%]; *P* < .001).

Having a caregiver with a higher risk of depression (CES-D score >16) was associated with worse QOL as measured by the PDQ-39 (mean [SD] score, 33.78 [17.71], on a scale of 0 to 60 with higher scores indicating greater risk of depression, among patients with caregivers who had depression symptoms vs 24.50 [14.19] among patients with caregivers who did not have depression symptoms; β = 6.89; 95% CI, 4.09-9.69; *P* < .001); this same association occurred when the CES-D score was treated as a continuous variable (β = 0.43; 95% CI, 0.28-0.58; *P* < .001) (Table 2). Furthermore, having a caregiver who reported greater depression symptoms was associated with an increase in ED visits (β = 0.02; 95% CI, 0-0.04; *P* = .03) when CES-D score was treated as a continuous variable. However, no association was observed when using the CES-D score cutoff of 16 (β = 0.15; 95% CI, −0.18 to 0.48; *P* = .38). There was no association between depression scores and hospitalizations when using the CES-D score cutoff of 16 (β = 0.20; 95% CI, −0.13 to 0.54; *P* = .24) or when treating the CES-D score as a continuous variable (β = 0.02; 95% CI, −0.01 to 0.03; *P* = .10).

**Table 2.** Association Between Caregiver Depression Symptoms and Patient Quality of Life, Hospitalizations, and Emergency Department Visits

Patient outcome	Mean (SD)		Association of depression symptoms with outcome, β (95% CI)	*P* value
	Caregiver without higher risk of depression^a^	Caregiver with higher risk of depression^b^		
**QOL (PDQ-39 score)** ^c^				
Caregiver CES-D total score	NA	NA	0.43 (0.28 to 0.58)^d^	<.001
Caregiver CES-D score >16	24.50 (14.19)	33.78 (17.71)	6.89 (4.09 to 9.69)^d^	<.001
**No. of annual hospitalizations**				
Caregiver CES-D total score	NA	NA	0.02 (−0.01 to 0.03)^e^	.10
Caregiver CES-D score >16	0.34 (0.84)	0.47 (0.97)	0.20 (−0.13 to 0.54)^e^	.24
**No. of annual ED visits**				
Caregiver CES-D total score	NA	NA	0.02 (0 to 0.04)^e^	.03
Caregiver CES-D score >16	0.45 (1.00)	0.66 (1.25)	0.15 (−0.18 to 0.48)^e^	.38

Abbreviations: CES-D, Center for Epidemiological Studies Depression Scale; ED, emergency department; NA, not applicable; PDQ-39, 39-item Parkinson Disease Questionnaire; QOL, quality of life.

^a^ CES-D score of 16 or lower on a scale of 0 to 60.

^b^ CES-D score of higher than 16 on a scale of 0 to 60.

^c^ Score range, 0 to 100, with higher scores indicating worse health-related QOL.

^d^ Mixed-effects linear model adjusted for patient age, sex, financial status, disease duration, Hoehn and Yahr stage, verbal fluency, delayed 5-word recall, caregiver self-reported health, and patient health (random effect).

^e^ Mixed-effects Poisson model adjusted for patient age, sex, financial status, disease duration, Hoehn and Yahr stage, verbal fluency, delayed 5-word recall, caregiver self-reported health, and patient health (random effect).

To contextualize these findings, we also compared the frequencies of total number of annual ED visits and hospitalizations longitudinally across all of the follow-up visit data points. In terms of annual ED visits, there were 686 patient-year data points among 978 total events (70.1%) during which no ED visits occurred in the cohort with caregivers who were not at higher risk of depression compared with 159 patient-year data points among 257 total events (61.9%) during which no ED visits occurred in the cohort with caregivers who were at higher risk of depression; overall, there was a statistically significant difference in annual ED visits per patient-year between the 2 groups (*P* = .004) (Table 3). With regard to annual hospitalizations, the cohort with caregivers who were not at higher risk of depression had 743 data points among 981 total events (75.7%) during which no hospitalizations occurred, whereas the cohort with caregivers who were at higher risk of depression had 180 data points among 258 total events (69.8%) during which no hospitalizations occurred (Table 3). This difference in the frequency of annual hospitalizations was also significant (*P* = .048).

**Table 3.** Frequency of Annual Emergency Department Visits and Hospitalizations by Caregiver Depression Symptoms Across All Follow-Up Visits

Outcome	Total annual events, No./total No. (%)		*P* value
	Caregiver without higher risk of depression^a^	Caregiver with higher risk of depression^b^	
Patient hospitalizations			
0	743/981 (75.7)	180/258 (69.8)	.048
1	183/981 (18.7)	56/258 (21.7)	
2	40/981 (4.1)	13/258 (5.0)	
3	9/981 (0.9)	3/258 (1.2)	
≥4	6/981 (0.6)	6/258 (2.3)	
Patient ED visits			
0	686/978 (70.1)	159/257 (61.9)	.004
1	208/978 (21.3)	63/257 (24.5)	
2	62/978 (6.3)	20/257 (7.8)	
3	9/978 (0.9)	8/257 (3.1)	
≥4	13/978 (1.3)	7/257 (2.7)	

Abbreviation: ED, emergency department.

^a^ Center for Epidemiological Studies Depression Scale score of 16 or lower on a scale of 0 to 60.

^b^ Center for Epidemiological Studies Depression Scale score of higher than 16 on a scale of 0 to 60.

## Discussion

This cohort study revealed several important findings regarding the association between caregiver depression and outcomes among patients with PD. Notably, patients with caregivers who had depression symptoms were more likely to have worse QOL and more likely to go the ED than those with caregivers who did not have depression symptoms.

Patients, families, clinicians, and policy makers increasingly recognize the importance of patient-reported outcome measures, such as health-related QOL, to evaluate the success of treatment. The 21st Century Cures Act of 2016 and the Prescription Drug User Fee Act require that the patient perspective be included in the development of new therapies.^15^ Greater caregiver depression symptoms have been found to alter PDQ-39 scores to an extent similar to that of neurostimulation (mean change in score, 9.5 points),^16^ while a clinically meaningful change in PDQ-39 score is estimated to be approximately 4 to 5 points.^17^ Although we cannot assert that the caregivers in our study definitively had clinical depression, this association suggests that treatment of caregiver distress may improve patient outcomes.

Our results were also consistent with those of a previous study^18^ involving caregivers of patients with dementia, which found increased ED use among caregivers at higher risk of depression. Depression is a warning sign of increased caregiver burden, which can lead to caregiver burnout and potentially lower caregiver efficacy. Thus, it is possible that patients with PD who have caregivers at higher risk of depression may have worse outcomes because depression hinders caregivers’ capacity to fulfill their demanding daily tasks. There has been sparse examination of the association of caregiver well-being with health care use among patients with PD. However, these results provide a more comprehensive view of the factors associated with patient care and outcomes and warrant follow-up study.

This study’s findings suggest that systematic screening for caregiver depression and more support of caregivers is important for the health of patients with PD and their caregivers. Innovative approaches, such as interdisciplinary home visits and peer mentoring programs,^19^ cognitive behavioral therapy for caregivers,^20^ outpatient palliative care,^21^ and comprehensive knowledge and skill training,^22^ can successfully reduce caregiver strain. Further study of the implications of caregiver depression treatment could be beneficial for individual health and financial strain and has the potential to reduce health care use and costs.

### Limitations

This study has several limitations. There were missing follow-up data for several participants, which introduced a source of bias. The lack of association between caregiver depression symptoms and patient hospitalizations could be, in part, due to lack of power resulting from this shortage of longitudinal data.

In addition, caregivers only completed the CES-D at the baseline visit. More CES-D data points would have aided in our analyses, including the ability to assess reverse association (ie, whether more severe PD symptoms were associated with greater caregiver burden, stress, and strain). Although we controlled for common measures of PD severity, it is possible that confounding due to unmeasured factors played a role in the observed findings. Furthermore, because the CES-D is a self-report screening questionnaire, we cannot definitively assert that caregiver depression was present. The inclusion of more formal diagnostic tools for depression would have led to a more robust characterization of caregiver depression. Patients in the sample were also highly educated and had access to specialty movement disorder clinics, which limits the generalizability of this study for underserved populations. Given this fact, this investigation should be replicated within underserved populations because the consequences of caregiver depression for patient outcomes may be even greater due to lack of access to health care as well as other concomitant stressors.

## Conclusions

This cohort study found that patients with PD who had caregivers at higher risk of depression were more likely to have worse QOL and higher ED use than patients who had caregivers not at higher risk of depression. Additional caregiving resources and interventions to reduce depression symptoms among caregivers could potentially improve patient outcomes.

## Notes

Supplement.
Data Sharing Statement

## References

[1] Hassan A, Wu SS, Schmidt P, . What are the issues facing Parkinson’s disease patients at ten years of disease and beyond? data from the NPF-QII study. *Parkinsonism Relat Disord*. 2012;18(Suppl 3):S10-S14. 10.1016/j.parkreldis.2012.06.014 22776044

[2] Aarsland D, Marsh L, Schrag A. Neuropsychiatric symptoms in Parkinson’s disease. *Mov Disord*. 2009;24(15):2175-2186. 10.1002/mds.22589 PMC2787875 19768724

[3] Wolff JL, Roter DL. Family presence in routine medical visits: a meta-analytical review. Soc Sci Med. 2011;72(6):823-831. doi:10.1016/j.socscimed.2011.01.015 21353358 PMC3070824

[4] Aarsland D, Larsen JP, Tandberg E, Laake K. Predictors of nursing home placement in Parkinson’s disease: a population-based, prospective study. J Am Geriatr Soc. 2000;48(8):938-942. doi:10.1111/j.1532-5415.2000.tb06891.x 10968298

[5] Vossius C, Nilsen OB, Larsen JP. Parkinson’s disease and nursing home placement: the economic impact of the need for care. Eur J Neurol. 2009;16(2):194-200. doi:10.1111/j.1468-1331.2008.02380.x 19146640

[6] Mosley PE, Moodie R, Dissanayaka N. Caregiver burden in Parkinson disease: a critical review of recent literature. J Geriatr Psychiatry Neurol. 2017;30(5):235-252. doi:10.1177/0891988717720302 28743212

[7] Greenwell K, Gray WK, van Wersch A, van Schaik P, Walker R. Predictors of the psychosocial impact of being a carer of people living with Parkinson’s disease: a systematic review. Parkinsonism Relat Disord. 2015;21(1):1-11. doi:10.1016/j.parkreldis.2014.10.013 25457815

[8] Marras C, Rafferty M, Davis T, et al. Data now available: annual Parkinson’s Outcomes Project (POP). *Moving Along*. September 2022. Accessed June 23, 2023. https://www.movementdisorders.org/MDS/Moving-Along/2022-issue-3/POP-data

[9] Okun MS, Siderowf A, Nutt JG, . Piloting the NPF data-driven quality improvement initiative. Parkinsonism Relat Disord. 2010;16(8):517-521. doi:10.1016/j.parkreldis.2010.06.005 20609611

[10] von Elm E, Altman DG, Egger M, Pocock SJ, Gøtzsche PC, Vandenbroucke JP. The Strengthening the Reporting of Observational Studies in Epidemiology (STROBE) statement: guidelines for reporting observational studies. *Ann Intern Med*. 2007;147(8):573-577. 10.7326/0003-4819-147-8-200710160-00010 17938396

[11] Carer Inclusive Working Group for ISO 25551. Terminology related to caregiving: an informative guide to commonly used concepts, words and phrases. International Standards Organization. Accessed June 23, 2023. https://committee.iso.org/files/live/sites/tc314/files/Terminology%20related%20to%20caregiving%20free%20resource.pdf

[12] Peto V, Jenkinson C, Fitzpatrick R. PDQ-39: a review of the development, validation and application of a Parkinson’s disease quality of life questionnaire and its associated measures. *J Neurol*. 1998;245 (Suppl 1):S10-S14. doi:10.1007/PL00007730 9617716

[13] Goetz CG, Poewe W, Rascol O, ; Movement Disorder Society Task Force on Rating Scales for Parkinson’s Disease. Movement Disorder Society Task Force report on the Hoehn and Yahr staging scale: status and recommendations. Mov Disord. 2004;19(9):1020-1028. doi:10.1002/mds.20213 15372591

[14] Radloff LS. The CES-D scale: a self-report depression scale for research in the general population. Appl Psychol Meas. 1977;1(3):385-401. doi:10.1177/014662167700100306

[15] Food and Drug Administration; Center for Drug Evaluation and Research; Center for Biologics Evaluation and Research. Patient-focused drug development: collecting comprehensive and representative input. US Dept of Health and Human Services. June 2020. Accessed June 23, 2023. https://www.fda.gov/media/139088/download

[16] Deuschl G, Schade-Brittinger C, Krack P, ; German Parkinson Study Group, Neurostimulation Section. A randomized trial of deep-brain stimulation for Parkinson’s disease. N Engl J Med. 2006;355(9):896-908. doi:10.1056/NEJMoa060281 16943402

[17] Horváth K, Aschermann Z, Kovács M, . Changes in quality of life in Parkinson’s disease: how large must they be to be relevant? Neuroepidemiology. 2017;48(1-2):1-8. doi:10.1159/000455863 28161701

[18] Guterman EL, Allen IE, Josephson SA, . Association between caregiver depression and emergency department use among patients with dementia. JAMA Neurol. 2019;76(10):1166-1173. doi:10.1001/jamaneurol.2019.1820 31282955 PMC6618767

[19] Fleisher JE, Suresh M, Klostermann EC, . IN-HOME-PD caregivers: the effects of a combined home visit and peer mentoring intervention for caregivers of homebound individuals with advanced Parkinson’s disease. Parkinsonism Relat Disord. 2023;106:105222. doi:10.1016/j.parkreldis.2022.11.014 36446676 PMC9825655

[20] Secker DL, Brown RG. Cognitive behavioural therapy (CBT) for carers of patients with Parkinson’s disease: a preliminary randomised controlled trial. *J Neurol Neurosurg Psychiatry*. 2005;76(4):491-497. 10.1136/jnnp.2004.042291 PMC1739584 15774433

[21] Kluger BM, Miyasaki J, Katz M, . Comparison of integrated outpatient palliative care with standard care in patients with Parkinson disease and related disorders: a randomized clinical trial. JAMA Neurol. 2020;77(5):551-560. doi:10.1001/jamaneurol.2019.4992 32040141 PMC7042842

[22] A’Campo LEI, Wekking EM, Spliethoff-Kamminga NGA, Le Cessie S, Roos RAC. The benefits of a standardized patient education program for patients with Parkinson’s disease and their caregivers. *Parkinsonism Relat Disord*. 2010;16(2):89-95. 10.1016/j.parkreldis.2009.07.009 19674927

