# Supplementary material for: Association of Caregiver Depression Risk With Patient Outcomes in Parkinson Disease
Source: JAMA Netw Open. 2023 Aug 11;6(8):e2327485. doi: 10.1001/jamanetworkopen.2023.27485 (PMC10422183; doi:10.1001/jamanetworkopen.2023.27485)
Supplement: Supplement. — Data Sharing Statement [file jamanetwopen-e2327485-s001.pdf]

Data Sharing Statement

Rashid. Association of Caregiver Depression Risk With Patient Outcomes in Parkinson Disease. *JAMA Netw Open*. Published August 11, 2023.
doi:10.1001/jamanetworkopen.2023.27485

Data
Data available: Yes
Data types: Data dictionary, Deidentified participant data
How to access data: https://redcap.parkinson.org/surveys/?s=A3L8A3TNHHAPXDPP
When available: With publication

Supporting Documents
Document types: None

Additional Information
Who can access the data: researchers whose proposed use of the data has been approved
Types of analyses: Specified purpose
Mechanisms of data availability: Signed data access agreement
